# Cellular Distribution Pattern of tjp1 (ZO-1) in *Xenopus laevis* Oocytes Heterologously Expressing Claudins

**DOI:** 10.1007/s00232-022-00251-z

**Published:** 2022-06-23

**Authors:** Nora Brunner, Laura Stein, Salah Amasheh

**Affiliations:** Institute of Veterinary Physiology, Freie Universität Berlin, Oertzenweg 19b, 14163 Berlin, Germany

**Keywords:** Claudins, Zonula occludens 1, ZO-1, tjp1, *Xenopus* oocytes

## Abstract

Epithelial barriers constitute a fundamental requirement in every organism, as they allow the separation of different environments and set boundaries against noxious and other adverse effectors. In many inflammatory and degenerative diseases, epithelial barrier function is impaired because of a disturbance of the paracellular seal. Recently, the *Xenopus laevis* oocyte has been established as a heterologous expression model for the analysis of transmembrane tight junction protein interactions and is currently considered to be a suitable screening model for barrier effectors. A prerequisite for this application is a physiological anchoring of claudins to the cytoskeleton via the major scaffolding protein tjp1 (tight junction protein 1, ZO-1). We have analyzed the oocyte model with regard to the interaction of heterologously expressed claudins and tjp1. Our experiments have revealed endogenous tjp1 expression in protein and mRNA analyses of unfertilized *Xenopus laevis* oocytes expressing human claudin 1 (CLDN1) to claudin 5 (CLDN5). The amphibian cell model can therefore be used for the analysis of claudin interactions.

**Graphical Abstract:**

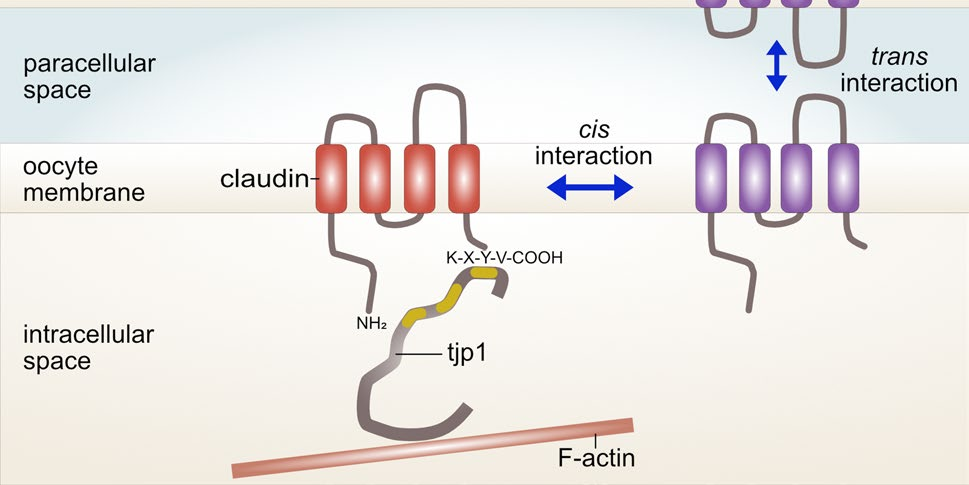

## Introduction

The epithelium acts as a biological, chemical, and physical barrier against multiple threats and challenges and provides a structural border between organ and tissue compartments (Powell 1981). The zonula occludens (tight junction, TJ), which is a complex intercellular junction, controls the permeability and transport of substances across the epithelium and is therefore indispensable for the physiology of the organism (Zihni et al. 2016). The tetraspan TJ protein family of claudins is the main determinant of organ- and tissue-specific TJs. Thus, detailed knowledge about claudin–claudin interactions is fundamental for the exertion of a suitable pharmacological influence on the barrier, because the paracellular seal is mainly provided by claudin–claudin protein interactions (Will et al. 2008).

The establishment of an alternative amphibian model system for barrier research has recently been described by our group (Vitzthum et al. 2019), which has shown that oocytes of the African claw frog *Xenopus laevis* can be employed for the analysis of claudin–claudin interactions. Recently, we have been able to expand this heterologous expression system to the blood–brain barrier protein CLDN5 and to extend the analytical approach by using hydrostatic pressure impulses for the further characterization of claudin *trans*-interactions (Brunner et al. 2020). Our current study focuses on fundamental aspects involved in the application of *Xenopus* oocytes for barrier research in the context of the cytoskeleton of the oocyte. Simultaneously with the establishment of the oocyte as a cell model for ion channel activity and transport mechanism, the cytoskeletal organization of the oocyte has been unveiled (Carotenuto and Tussellino 2018). As a result, a wide range of techniques had been established which allow a manipulation, disruption, and rigidization of the oocyte membrane. Some of the pharmacological strategies, e.g., the block of actin polymerization by cytochalasin D (Galizia et al. 2012, 2013) or the disruption of cytoplasmic structures by the emptied-out *Xenopus* oocyte technique (EOO) to test potential drug effects on the intracellular binding sites of the oocyte membrane (Ozu et al. 2005, 2011) may become relevant in the clinical implementation of claudin-expressing *Xenopus* oocytes, as well.

The major link between cytoskeletal actin filaments and tetraspan TJ proteins is provided by tjp1 (Furuse et al. 1994). Tjp1 is a cytoplasmic protein that contains PDZ-binding sites for barrier proteins including claudins (Furuse et al. 1998; Itoh et al. 1999), occludin (Furuse et al. 1994; Fanning et al. 1998), and tricellulin (Ikenouchi et al. 2005; Riazuddin et al. 2006). Further functions include binding to gene-regulating transcription factors, e.g., ZONAB (Balda and Matter 2000; Balda et al. 2003). Various alternative RNA splicing isoforms have been described for tjp1, namely a longer isoform with 80 extra amino acids (α^+^) and a shorter isoform lacking this alpha domain (α^−^) (Willott et al. 1992). Although tjp1 depletion has been shown to be lethal in mouse embryos (Katsuno et al. 2008), other authors have observed that claudins lacking the PDZ motif still localize to the TJ and can dynamically break and re-anneal into TJ strands (Ruffer and Gerke 2004; Van Itallie et al. 2017). Moreover, tjp1 plays a fundamental role in the kinetics of TJ assembly (Fanning and Anderson 2009) and has a stabilizing effect on the solute barrier through coupling to the cytoskeletal ring of the cells (Van Itallie et al. 2009). But also a manipulation through sense and antisense Shroom oligonucleotide injection as shown for xShroom1 has an impact on membrane protein function and maintenance mediated through the effects on amiloride-sensitive Na^+^ currents in *Xenopus* oocytes (Zuckerman et al. 1999; Assef et al. 2011; Palma et al. 2016). Many of these regulatory proteins do share similarities in domains with PDZ. In our current study, we present a first assessment of the localization of the heterologously expressed claudins and PDZ-containing tjp1, which is of major interest for the employment of the amphibian cell model in membrane barriology.

*Xenopus laevis* is a widely used model organism for developmental biology and translational research (Nenni et al. 2019), and thus, its genomic evolution and embryonic development have previously been described in detail (Bowes et al. 2008; Segerdell et al. 2008; Session et al. 2016). When *Xenopus* oocytes have been employed for the heterologous expression of proteins, unfertilized oocytes of stages V and VI have been used with a gene expression for *tjp1* S and for *tjp1* L of 0.9 transcripts per one million mapped reads (TPM) and of 1.9 TPM, respectively (Session et al. 2016). The relative protein expression for oocytes at stage VI is described as being 0.096, which represents the decimal fraction at this stage of total protein agglomerated over all profiled stages (Peshkin et al. 2019). In embryonic development, zygotic transcription starts from the 4000-cell stage onward (Fesenko et al. 2000), but TJs and associated structures can be observed from the (fertilized) 2-cell stage onward and are translated from maternal stores of mRNA (Cardellini et al. 1996; Heasman 2006).

An investigation of the influence of endogenous tjp1 expression on claudin-expressing oocytes and an evaluation of the functionality of the protein–scaffold interaction are essential requirements for further application of *Xenopus* oocytes in the context of barrier research. In this study, we have screened *Xenopus* laevis oocytes for their endogenous expression and localization of tjp1 protein in context with heterologous claudin expression. Additionally, we have analyzed possible claudin-specific regulatory effects on *tjp1* gene expression.

## Materials and Methods

### Animals

Oocytes were obtained from mature female African claw frogs. Animal treatments were conducted with approval by the animal welfare officer for the Freie Universität Berlin and under the governance of the Berlin Veterinary Health Inspectorate (Landesamt für Gesundheit und Soziales Berlin, permit O 0022/21).

### Anesthetics and Surgical Procedure

To achieve surgical anesthesia of the frogs, they were transferred into a bath solution of buffered 2 g/L MS222 (ethyl 3-aminobenzoate methanesulfonate, Sigma-Aldrich, Taufkirchen, Germany, pH 7.5) for 5–10 min at 20 °C. Righting and corneal reflexes were used for the assessment of surgical anesthetic depth. Skin and abdominal muscle incisions were made to access the *Xenopus* ovaries.

### cRNA Preparation

Relevant nucleotide coding consensus sequences were used for the synthesis of the human cRNA of CLDN1 to CLDN5 (ShineGene Bio-Technologies Inc., Shanghai, China; Thermo Fischer Scientific, Henningsdorf, Germany). Claudin sequences were cloned into suitable high copy ampicillin-resistant pGEM for transformation in competent DH10b *Escherichia coli*. A commercial T7 RNA-polymerase-based approach (T7 RiboMAX RNA Production System and Ribo m^7^G Cap Analog, Promega, Walldorf, Germany) was used according to the manufacturer’s instructions to generate cRNAs for injection into the amphibian germ cells.

### Oocyte Isolation and cRNA Injection

Follicular cell layers were removed by enzymatic digestion at room temperature for 90 min in 1.5 mg/ml collagenase (NB4 Standard Grade, Nordmark Pharma, Germany) dissolved in oocyte Ringer solution (ORi). Cells were then separated by incubation in Ca^2+^-free ORi (Vitzthum et al. 2019) for 10 min on a mechanical shaker at 50 rpm. Oocyte stages V and VI were injected (Nanoliter 2010, World Precision Instruments, Sarasota, USA) with 50.6 nl of 10 ng/µl, 20 ng/µl, or 40 ng/µl cRNA encoding for human CLDN1 to CLDN5 or with RNase-free water as controls. Based on the total cRNA amounts, this gave three experimental groups: 0.5, 1, and 2 ng cRNA/oocyte. Injected oocytes were incubated for 3 days at 16 °C in ORi for protein expression.

### Isolation of Membrane Fractions and Immunoblotting

For Western blot analysis, ten injected oocytes were blended and resuspended in 500 µl oocyte homogenization buffer containing (in mM) 5 MgCl_2_, 5 NaH_2_PO_4_, 1 EDTA, 80 sucrose, and 20 Tris, pH 7.4 in accordance with the plasma membrane buffer established by Leduc-Nadeau et al. (Leduc-Nadeau et al. 2007). Oocyte suspensions were centrifuged twice at 200 rpm for 10 min at 4 °C, and the supernatant was centrifuged at 13,000 rpm for 30 min at 4 °C. The pelletized cell membrane fractions were resuspended in homogenization buffer. Membrane samples were then quantified with Protein Bioassay according to the manufacturer´s instruction in a 96-well plate (#500-0119 RC DC Protein Assay, Bio-Rad, Munich, Germany). Bovine Serum Albumin Standard (ThermoFischer Scientific, Henningsdorf, Germany) served as the protein standard. Before the loading of the gels, samples were mixed with 4× Laemmli buffer (Bio-Rad Laboratories, Munich, Germany). Samples were loaded onto a stain-free acrylamide gel (TGX Stain-Free FastCast Acrylamide Kit, 10% #1610183, Bio-Rad Laboratories, Munich, Germany) and electrophoresed. The proteins were transferred to PVDF membranes, and the binding of nonspecific proteins was blocked with 5% nonfat dry milk in Tris-buffered saline for 60 min. We detected the proteins of interest by incubation of the membranes with primary antibodies raised against the TJ proteins CLDN1 to CLDN5 and tjp1 (#51-9000, #51-61600, #35-2500, #32-9400, #34-1700, Life Technologies, Carlsbad, USA, and LS-C145545-100, Biozol, Eching, Germany) overnight at 4 °C. Peroxidase-conjugated secondary antibodies (#7074, #7076 Cell Signaling Technology, Danvers, MA, USA) were incubated with the membranes for 45 min at room temperature and detected using Clarity Western ECL Blotting Substrate and ChemiDoc MP (#1705061, Bio-Rad Laboratories GmbH, Munich, Germany).

### Immunofluorescence Cytochemistry

Using our established protocols, oocytes were paired for the analysis of claudin *trans*-interactions (Brunner et al. 2020). Briefly, vitelline membranes were removed, and claudin-expressing oocytes were clustered to induce adhering contact areas. Oocyte pairs were incubated in ORi at 16 °C for 24 h. Oocytes were fixed in 4% PFA (16% paraformaldehyde, E15700, Science Service, Munich, Germany) for 4 h at room temperature followed by dehydration in an alcohol gradient to xylol. Samples were embedded in paraffin, cross-sectioned (5 µm), and mounted onto microscope slides.

Primary antibodies were the same as those for immunoblotting, and secondary antibodies were conjugated with photostable Alexa Fluor 488 and Alexa Fluor 594 dyes (Life Technologies, Carlsbad, USA). Slides were examined by confocal laser-scanning immunofluorescence microscopy (Zeiss LSM 710).

### RNA Isolation and cDNA Synthesis

The Nucleospin RNA (Macherey & Nagel, Dueren, Germany) commercial kit was used for RNA extraction from 10 oocytes per sample. NanoPhotometer P330 (Implen GmbH, Munich, Germany) was employed to determine the levels of possible contamination. An RNA absorption ratio of light at 260/280 nm > 2 was considered to indicate that the samples were free of protein contamination. A 260/230 nm absorption ratio of 1.7–2 was considered to indicate that the samples were free of buffer salt contamination.

cDNA was synthesized using iScript (Bio-Rad, Munich, Germany) according to the manufacturer’s instructions. A–RT sample (without reverse transcriptase) was used as a negative running control. For reverse transcription, a Biorad iCycler iQTM (Biorad, USA) was used with the protocol given in Table 1.

**Table 1:** Reverse transcription protocol

	Time (min)	Temperature (°C)
Priming	5	25
Reverse transcription	30	42
Inactivation of cDNA	5	85

### Qualitative and Quantitative Real-Time PCR

For PCR analysis, *Xenopus laevis odc1* (ornithine decarboxylase 1), *gapdh* (glyceraldehyde-3-phosphate dehydrogenase), and *h4c4* (H4 clustered histone 4) were used as housekeeping genes, and *tjp1* as the gene of interest. Primers (Table 2) were purchased from Eurofins Genomics (Eurofins, Ebersberg, Germany). For qualitative PCR, cDNA samples from claudin-injected oocytes were pooled and transcribed using *Taq* PCR master mix (Qiagen, *#*201443, Düsseldorf, Germany) according to the instructions of the manufacturer. Following gene amplification (Table 3), PCR products were loaded onto a 2% agarose gel in TBE buffer. Additionally, quantitative PCR was performed using iQTM SYBR Green Supermix Kit (Biorad, USA) with three replicates per reaction and three technical replicates. Double-distilled H_2_O and –RT samples served as negative controls. As primer efficiency ranged between 1.93 and 2.03, gene expression was normalized relative to the housekeeping genes and to the control group by using the Delta–Delta CT method.

**Table 2:** Primers for qPCR

	Amplicon length (bp)	Sense sequence	Antisense sequence
*odc1*	221	GCCATTGTGAAGACTCTCTCCATTC	TTCGGGTGATTCCTTGCC
*gapdh*	201	CTCTCGCAAAGGTCATCAA	CGTTCAGCTCAGGGATAAC
*h4c4*	103	GACGCTGTCACCTACACCGAG	CGCCGAAGCCGTAGAGAGTG
*tjp1*	205	GGACAGAAGTTTATCACCAAGA	CTTAAGCACCACGTCTCC

**Table 3:** PCR protocols for *tjp1* gene expression analysis

	Qualitative PCR		Quantitative PCR	
	Time	Temperature	Time	Temperature
Initial denaturation and polymerase activation	3 min	94 °C	3 min	95 °C
Denaturation	30 s	94 °C	12 s	95 °C
Annealing and extension	1 min	57 °C	1 min	60 °C
40 cycles				

### Statistical Analysis

Statistical analysis was performed with JMP Pro 15.0.0 (NC, USA). The normal distribution was checked using the Shapiro–Wilk test, and Delta CT values were analyzed by one-way analysis of variance (ANOVA).

## Results

### Heterologous Expression of TJ Proteins in *Xenopus* oocytes

**Fig. 1:**
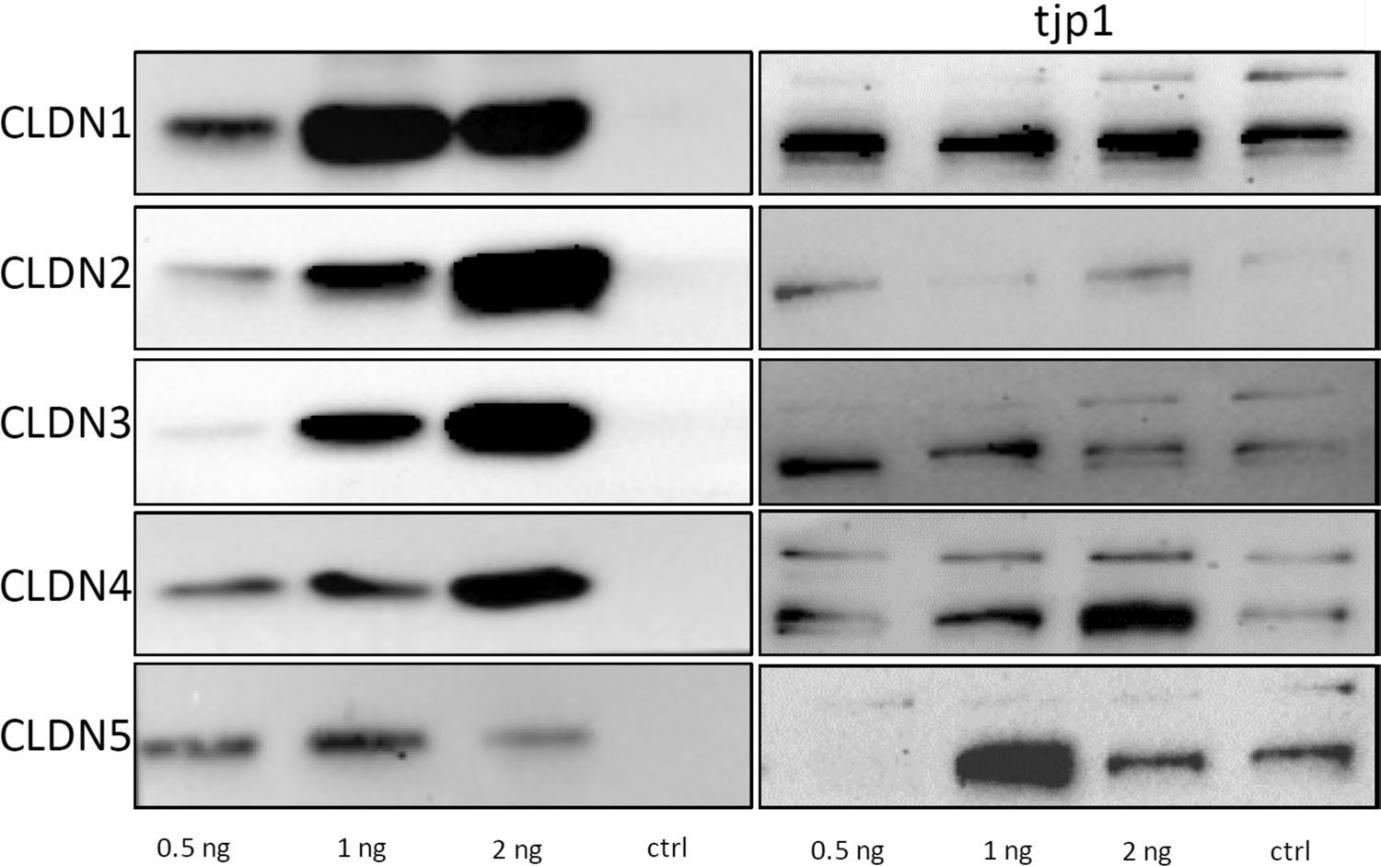
Immunoblot analysis of tight junction (TJ) proteins in *X. laevis* oocytes Cell membrane lysates applied to 10% stain-free acrylamide gel and transferred onto PVDF membranes. All claudin-injected oocytes membranes revealed claudin-specific signals at the predicted protein mass in accordance with the injected cRNAs (20–27 kDa). RNAse-free water-injected oocytes were treated identically and showed no signal for endogenous expression of claudins. However, specific signals for both tjp1 isoforms α^+^ (195 kDa) and α^−^ (187 kDa) in claudin-expressing oocytes and water-injected controls confirmed endogenous tjp1 protein expression

The successful expression and integration of claudins into the *Xenopus laevis* plasma membrane was verified by Western blot analysis. After 3 days of expression, membrane fractions of 10 oocytes having had injections of 0.5 ng/oocyte, 1 ng/oocyte, or 2 ng/oocyte claudin cRNA were loaded onto a stain-free acrylamide gel. All membranes revealed claudin-specific signals at the predicted protein mass in accordance with the injected cRNAs (20–27 kDa). RNAse-free water-injected oocytes were treated identically and showed no signal for the endogenous expression of claudins (Fig. 1). Samples were also incubated with tjp1 antibody to check the endogenous tjp1 expression in the claudin-injected cells. All tested oocytes showed tjp1 isoform-specific signals at 187 kDa and 195 kDa.

### Oocytes Show Specific Signals of tjp1 in the Submembranous Space

**Fig. 2:**
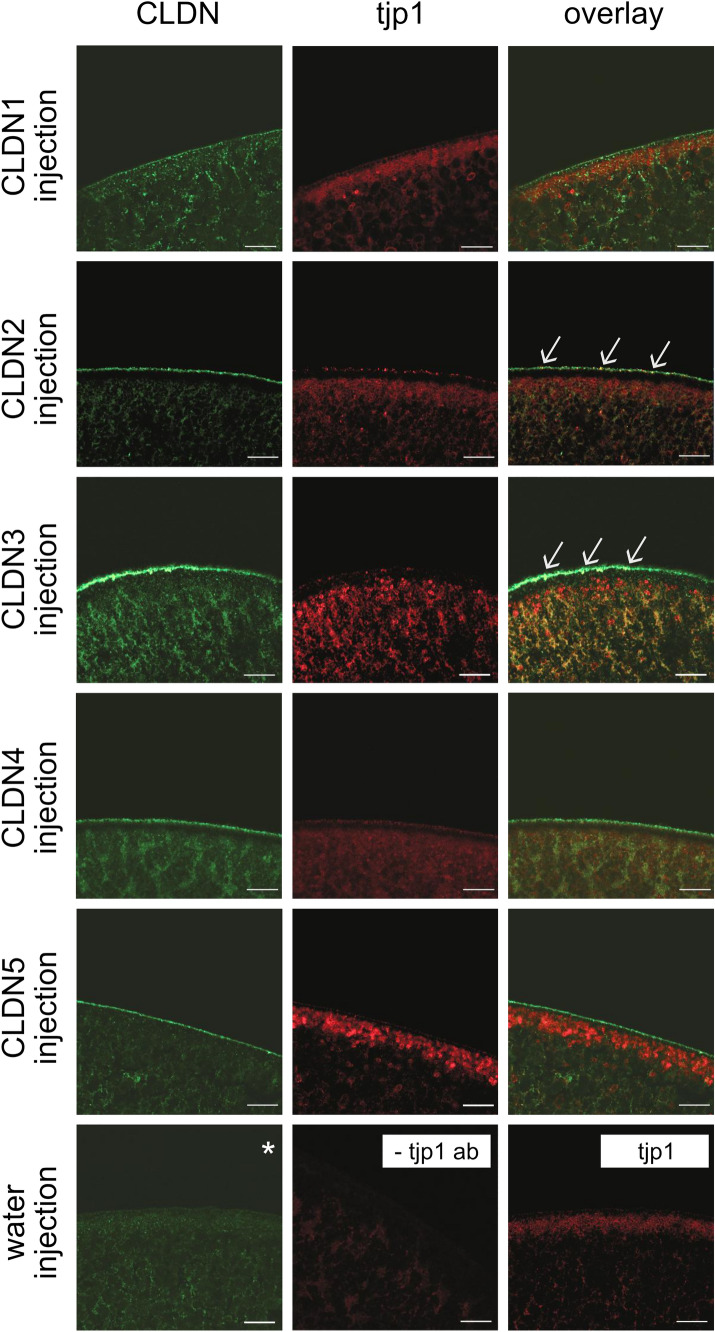
Immunohistochemical staining of TJ proteins in *X. laevis* oocytes All claudin-injected oocytes revealed claudin-specific signals at their cell membranes in accordance with the injected CLDN cRNAs (green). RNAse-free water-injected oocytes were treated identically and showed no signal for the endogenous expression of claudins (* representative image of water-injected oocyte screened for endogenous CLDN3 expression). Additionally, immunofluorescent staining in claudin- and water-injected oocytes revealed specific tjp1 signals (red) in oocytes, whereas in no primary antibody controls, no specific signals were detected by confocal microscopy. Tjp1 signals were concentrated in the submembranous space and appeared as a belt-like structure. In CLDN2- and CLDN3-expressing oocytes, claudin and tjp1 signals were selectively colocalized at the plasma membrane and resulted in a yellow signal (arrows). Scale bars: 20 mm

After removal of vitelline membranes, claudin-expressing and water-injected control oocytes were clustered into pairs. Both control and claudin-injected oocytes showed specific immunohistochemical signals after incubation with tjp1 antibodies. The signal was mainly located in the submembranous space of the cells and appeared as a submembranous belt immediately underneath the oocyte plasma membrane (Fig. 2). This accumulation of signals was particularly distinct in the CLDN1-, CLDN2-, and CLDN5-expressing cells and in naïve oocytes. In CLDN2- and CLDN3-expressing cells, claudin and tjp1 signals were selectively colocalized at the plasma membrane and resulted in a yellow signal (arrows).

### Claudin Injection Does Not Engage Endogenous tjp1 mRNA Expression

**Fig. 3:**
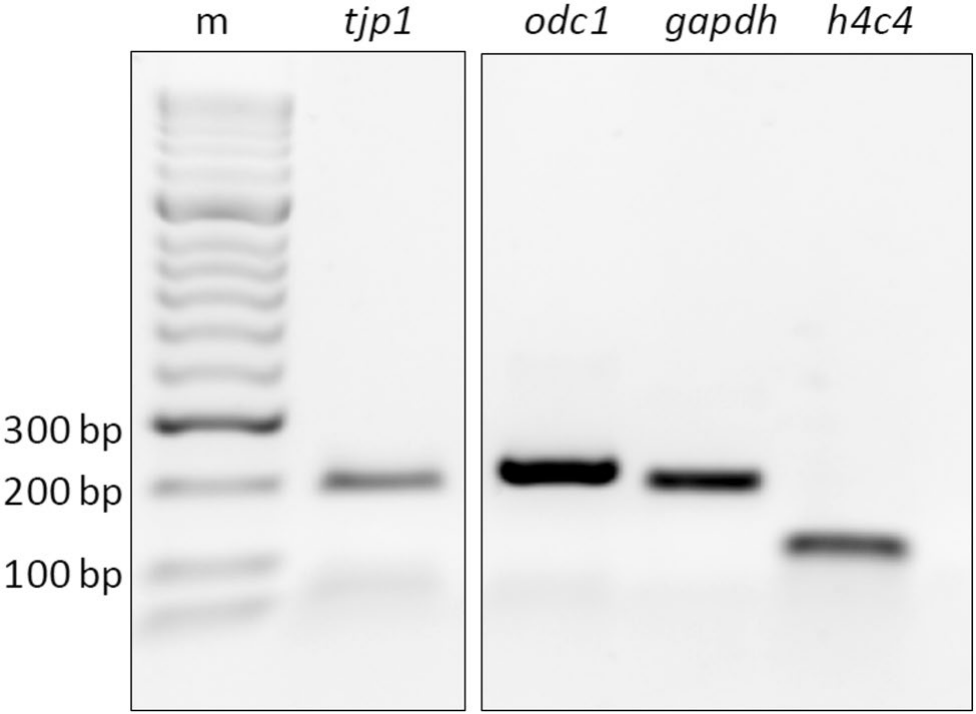
Qualitative PCR of *tjp1* and housekeeping genes *odc1*, *gapdh*, and *h4c4* PCR products were loaded onto a 2% agarose gel in TBE buffer. Pooled samples of claudin-injected oocytes showed gene products in accordance with the predicted amplicon size (Table 2) of the housekeeping genes and the gene of interest

**Fig. 4:**
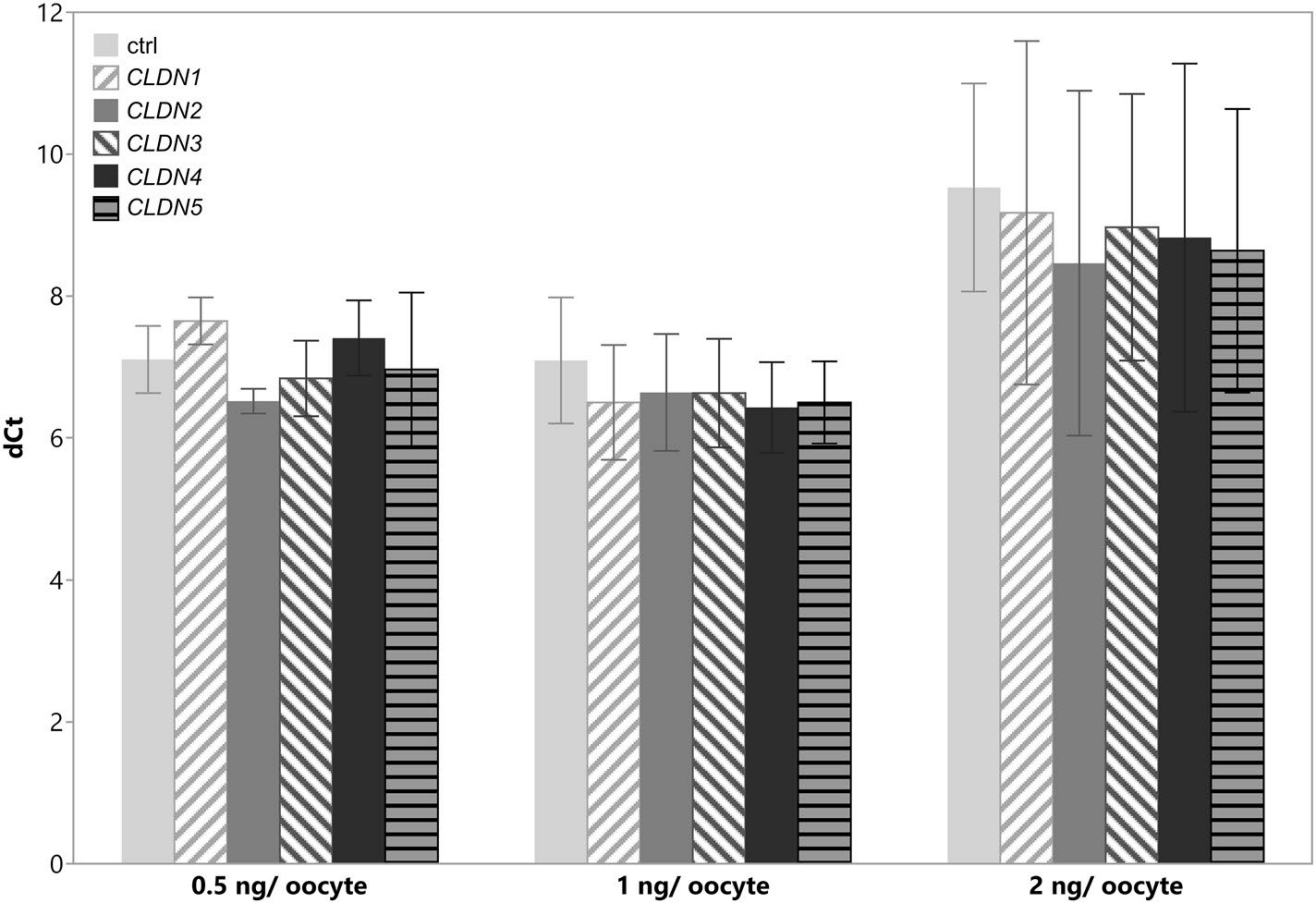
Quantitative real-time PCR of *tjp1* in claudin-expressing *X. laevis* oocytes PCR delta Ct values in control oocytes were indistinctive from claudin-expressing oocytes at all three tested concentrations of 0.5, 1, and 2 ng cRNA/ oocyte, ANOVA: *p* ≥ 0.9

*Tjp1* was consistently detectable by qualitative PCR (Fig. 3). We therefore performed quantitative real-time PCR to investigate the effect of claudin injection on *tjp1* mRNA levels. Delta CT values were analyzed for all three concentrations by one-way analysis of variance (ANOVA) to determine the effect of claudin injection and water-injected controls, *F* (5, 48) = 0.2367, *p* ≥ 0.9). All claudin-injected oocytes showed a negligible impact of the claudin injection on *tjp1* expression compared with water-injected control oocytes, resulting in a mild n-fold upregulating trend of 1.28–2.10 for *tjp1* expression in claudin-expressing cells (not significant; Table 4 and Fig. 4).

**Table 4:** Delta Ct and *n*-fold *tjp1* expression

Delta Ct ± SEM				*n*-fold (control = 1)		
	0.5 ng/oocyte	1 ng/oocyte	2 ng/oocyte	0.5 ng/oocyte	1 ng/oocyte	2 ng/oocyte
Control	7.105 ± 0.223	7.090 ± 0.419	9.530 ± 0.692			
CLDN1	7.647 ± 0.156	6.499 ± 0.382	9.174 ± 1.142	1.28	1.51	1.28
CLDN2	6.517 ± 0.082	6.640 ± 0.389	8.462 ± 1.146	2.10	1.37	2.10
CLDN3	6.838 ± 0.251	6.629 ± 0.361	8.970 ± 0.886	1.47	1.38	1.47
CLDN4	7.406 ± 0.250	6.428 ± 0.301	8.825 ± 1.157	1.63	1.58	1.63
CLDN5	6.964 ± 0.511	6.499 ± 0.273	8.639 ± 0.693	1.85	1.51	1.85

## Discussion

In our present study, we have further characterized the established heterologous expression system of *Xenopus* oocytes for the analysis of barrier proteins (Vitzthum et al. 2019). As an interplay between the cytoskeletal scaffold and the expressed barrier proteins provides the foundation of physiological barrier formation (Rodgers et al. 2013), the investigation of interactions between these proteins in *Xenopus* oocytes appears mandatory for further applications of the model system.

We employed immunoblotting and immunohistochemical staining in order to gain a comprehensive understanding of the expression, localization, and interaction of heterologously expressed claudins with tjp1 in oocytes at developmental stages V and VI. *Xenopus* oocytes at stage V–VI express small amounts of transcripts of claudin mRNA, ranging from approximately 0.06 up to 44.7 TPM (Session et al. 2016), and so, endogenous claudin protein expression might be expected in immunoblots. But the protein expression of claudins is described as a mere fraction, e.g., 0.001 for cldn3 (decimal fraction at stage VI of total protein agglomerated over all profiled stages), and the anti-human CLDN antibodies allowed a clear distinction to be made between injected and thus overexpressing oocytes and naïve germ cells. Nevertheless, we were able to verify endogenous tjp1 protein expression and to localize the protein to the submembranous space of naïve and claudin-expressing oocytes. In accordance with the literature in which both isoforms of tjp1 have been reported to be present in the *Xenopus* embryo from the first cleavage onwards (Fesenko et al. 2000), we were able to detect α^+^ and α^−^ tjp1 in oocytes at stages V and VI.

Furthermore, our quantitative PCR analyses revealed that claudin expression did not significantly affect *tjp1* mRNA expression levels. Previously, tjp1 has been shown to have a modeling effect on cell–cell contacts by regulating nuclear processes (Gottardi et al. 1996). In addition, claudins have been described as transcriptional regulators (Hagen 2017) that not only affect other transcription factors, e.g., ZONAB (Ikari et al. 2014), but also have the ability to interact with the scaffold. Schlingmann et al. have demonstrated that the binding of CLDN5 to tjp1 in alveolar epithelial cells results in paracellular leakage and the rearrangement of TJs by inhibiting the interaction of CLDN19 with the scaffold (Schlingmann et al. 2016). Moreover, a reduction in tjp1 to CLDN4 binding has been shown to lead to lower CLDN4 expression (Hamada et al. 2013). In our study, heterologous claudin expression did not affect *tjp1* gene expression in the oocytes, and claudin–scaffold interactions were reflected by only a partial colocalization of the two binding partners (CLDN and tjp1) at the same intracellular location, as shown by confocal laser-scanning analyses. Unlike the localization in epithelial cells and cell culture experiments in which tjp1 and claudins largely colocalize in the apical part of the cells, the clear distinction between the membranous claudins and the submembranous scaffolding protein tjp1 becomes more apparent, because of the large size of the germ cell of up to 1300 µm. Nevertheless, the limited counts of colocalization indicate that claudins and tjp1 are only intermittently associated corresponding to the dynamic coupling of claudin strands with the cytoskeleton (Van Itallie et al. 2017). In the literature, actin filaments of oocyte stage VI have been observed to surround the germinal vesicle and also extend from the cortex into the subcortical cytoplasm. After this stage, a dynamic change of actin distribution has only been described after the meiotic arrest of prophase I is terminated and fertilization occurs (Roeder and Gard 1994; Christensen et al. 1984). Furthermore, independent of the interaction with tjp1 or actin, claudin strands are capable to break and re-anneal (Van Itallie et al. 2017), although the accumulation of the tjp1 signal in the submembranous space is described as an indicator of the formation of the subjunctional cytoplasmic plaque of the TJ (D'Atri and Citi 2002). The accumulation of tjp1 in a submembranous belt in oocytes resembles the concentration of tjp1 in the junctional complex region in polarized epithelial cell lines (Umeda et al. 2006), and thus, the formation of the submembranous belt in *Xenopus* oocytes might mirror this process of organization. We conclude that, in this experimental setting, physiological binding is unhampered. The reason that the submembranous signal is more apparent in CLDN1-, CLDN2-, and CLDN5-expressing cells compared with CLDN3- and CLDN4-expressing cells remains unclear and needs to be examined in more detail in future studies. An overexpression of CLDN3 and CLDN4 has been described to enhance tumorigenesis of human ovarian surface epithelial (HOSE) cells. The more diffuse pattern of tjp1 in the oocytes might therefore result from tjp1 interacting with not only claudins, but also numerous other cytosolic and nuclear proteins, e.g., pten and zonab, which play a role in the regulation of germ cell function (Heinzelmann-Schwarz et al. 2004; Agarwal et al. 2005). Additionally, Nomme et al. have identified factors of claudin specificity and affinity of binding to cytoplasmic scaffolding proteins, such as tjp1. They analyzed the binding of claudins to the tjp1 PDZ1 domain and discovered that the binding can be influenced by the presence or absence of a tyrosine residue at P_-6_ and that the affinity is reduced if the tyrosine is modified by phosphorylation (Nomme et al. 2015). However, these findings can not depict a full molecular explanation for the structural distinct cellular localization of tjp1 in the *Xenopus* oocytes, because CLDN1 and CLDN4 do not share this tyrosine residue at P_-6_. Moreover, a potential difference might arise because of a disparate distribution of yolk platelets along the animal–vegetal axis of the oocytes (Danilchik and Gerhart 1987), rather than because of differences with regard to claudin family members.

Although *Xenopus laevis* is widely used for the investigation of transport mechanisms, signaling pathways, and human hereditary genetic diseases (Miller and Zhou 2000; Blum et al. 2009; Blum and Ott 2018), the use of *Xenopus* oocytes for barrier research is a novel approach. Two studies have recently been conducted on the mechanistic suitability of the oocytes for barriology by our group (Vitzthum et al. 2019; Brunner et al. 2020). The current study contributes to this specific field of barrier research and encourages the application of the model. Despite the information that a single or two-cell (paired oocyte) model can contribute to a multifunctional and multicellular barrier system being limited, it nevertheless allows an in-depth examination of claudin interaction in a restricted and therefore verifiable, reproducible, and cost-efficient model system.

In our experimental setup, the effects of claudin expression on the cytoskeletal scaffold are demonstrated for tjp1. In a further step toward a better understanding of tjp1-CLDN colocalization, Förster resonance energy transfer (FRET) technology or coimmunoprecipitation (coIP) could be conducted in follow-up studies to gain a sterical perception of the involved mechanism and give proof of an interaction between the binding partners. In particular, the detection of small quantities of endogenous tjp1 in *Xenopus* oocytes might be improved as it was shown for cystic fibrosis transmembrane regulator (CFTR) protein localization by Kreda et al. (Kreda and Gentzsch 2011). Additionally, a coinjection of CLDNs and tjp1 cRNA leading to a tjp1 overexpression may lead to further insights into the tjp1-CLDN interaction and might also allow a manipulation of CLDN function through the utilization of tjp1 orthologs and mutants. This might further allow clinical implications, toward an understanding and therapeutical options including the role of the actin cytoskeletal scaffold in barrier-related diseases, e.g., IBD (Kuo et al. 2021).

Although tjp1 plays an important role with regard to TJ assembly, structure, and regulation, the development of a functional barrier is dependent on a variety of factors, such as MARVEL domain proteins (Raleigh et al. 2010), junctional adhesion molecules, and cingulin (D’Atri et al. 2002; Zihni et al. 2016; Vasileva et al. 2020). Indeed, tjp1 can be regarded as a key point of TJ scaffolding, as reduced tjp1 expression correlates with increased TJ permeability and ineffective epithelial healing processes (Kuo et al. 2021). Thus, our present examination of tjp1-CLDN interactions provides a timely evaluation of the accessibility of the amphibian cell model for barrier research.

## Data Availability

In accordance with the rules of good scientific practice, all data are archived and available on request.

## Abbreviations

TJ: Tight junction
CLDN: Claudin
tjp1, ZO-1: Tight junction protein 1
